# A metabolic intermediate of the fructose-asparagine utilization pathway inhibits growth of a *Salmonella fraB* mutant

**DOI:** 10.1038/srep28117

**Published:** 2016-07-12

**Authors:** Anice Sabag-Daigle, Henry M. Blunk, Anindita Sengupta, Jikang Wu, Alexander J. Bogard, Mohamed M. Ali, Christopher Stahl, Vicki H. Wysocki, Venkat Gopalan, Edward J. Behrman, Brian M. M. Ahmer

**Affiliations:** 1 Department of Microbial Infection and Immunity, The Ohio State University, Columbus, OH 43210, USA; 2 Center for Microbial Interface Biology, The Ohio State University, Columbus, OH 43210, USA; 3 Department of Microbiology, The Ohio State University, Columbus, OH 43210, USA; 4 Department of Chemistry and Biochemistry, The Ohio State University, Columbus, OH 43210, USA; 5 Department of Medical Microbiology and Immunology, Faculty of Medicine, Mansoura University, Mansoura, Egypt

## Abstract

Insertions in the *Salmonella enterica fra* locus, which encodes the fructose-asparagine (F-Asn) utilization pathway, are highly attenuated in mouse models of inflammation (>1000-fold competitive index). Here, we report that F-Asn is bacteriostatic to a *fraB* mutant (IC_50_ 19 μM), but not to the wild-type or a *fra* island deletion mutant. We hypothesized that the presence of FraD kinase and absence of FraB deglycase causes build-up of a toxic metabolite: 6-phosphofructose-aspartate (6-P-F-Asp). We used biochemical assays to assess FraB and FraD activities, and mass spectrometry to confirm that the *fraB* mutant accumulates 6-P-F-Asp. These results, together with our finding that mutants lacking *fraD* or the *fra* island are not attenuated in mice, suggest that the extreme attenuation of a *fraB* mutant stems from 6-P-F-Asp toxicity. *Salmonella* FraB is therefore an excellent drug target, a prospect strengthened by the absence of the *fra* locus in most of the gut microbiota.

There are over 2600 serovars of *Salmonella* that can be divided into two pathovars, the gastrointestinal pathovar that causes inflammatory diarrhea, and the extraintestinal pathovar that typically fails to elicit diarrhea but instead causes a systemic infection, Typhoid Fever^1^. *Salmonella enterica* serovar Typhimurium is the model gastrointestinal serovar, while serovar Typhi is the classic example of an extraintestinal serovar. The gastrointestinal serovars of *Salmonella* are among the most common of foodborne illnesses in the United States, and are the leading cause of hospitalization and death^2^. Globally, the gastrointestinal serovars are thought to cause 155,000 deaths per year^3^,^4^. There are no vaccines for human use that protect against the gastrointestinal serovars^5^,^6^. Antibiotics are used to treat the very young or elderly, or when there are complications or systemic disease. However, for uncomplicated cases of *Salmonella*-mediated gastroenteritis, antibiotics are not recommended because the disruption to the normal microbiota may actually benefit *Salmonella* and increase shedding^7^,^8^,^9^. Therefore, drugs that specifically target *Salmonella* without disrupting the microbiota would be extremely useful. Focusing on the unique metabolic capabilities of this pathogen is likely to prove productive.

Serovar Typhimurium causes inflammation using its two Type 3 Secretion Systems, T3SS1 and T3SS2, encoded within *Salmonella* Pathogenicity Islands 1 and 2 (SPI1 and SPI2), respectively^10^. This inflammation, which disrupts the microbiota and presumably removes competitors for nutrients^11^, leads to the oxidation of thiosulfate to tetrathionate that can be used by *Salmonella* as a respiratory electron acceptor^12^. *Salmonella* then respires while its competitors ferment, allowing *Salmonella* to grow rapidly^13^,^14^. Respiration also increases the number of compounds that can be utilized as nutrients, as some (e.g., ethanolamine) can be respired but not fermented^15^.

We recently reported that *Salmonella* can utilize fructose-asparagine (F-Asn) as a sole carbon and nitrogen source^16^. A *fraB*::kan mutant of *Salmonella* was not able to utilize F-Asn and was extremely attenuated in mouse models of inflammation^16^. *fraB* is the first gene of the putative *fraBDAE* operon. The *fraR* gene, which encodes a transcription factor of the GntR family, is located upstream of *fraBDAE* and oriented in the same direction. These five genes make up a horizontally acquired island that is not present in *Escherichia coli*. The proposed metabolic pathway catalyzed by these gene products is shown in Fig. 1. In this report, we mutated the four structural genes and found that each plays a role in F-Asn utilization, although the *fraE* mutation did not completely eliminate F-Asn utilization. During these studies we determined that F-Asn is inhibitory to a *fraB* mutant, but not to the other mutants, and determined that a metabolic intermediate accumulates in the *fraB* mutant. We found that this inhibition is responsible for the extreme attenuation in mouse models, and identify FraB as a novel drug target.

## Results

### A *fraB* mutant of *Salmonella* cannot grow on glucose in the presence of F-Asn

Wild-type *Salmonella* and an isogenic *fraB* mutant grow equally well on glucose as sole carbon source (Fig. 2). The wild-type grows on F-Asn as the sole carbon source, while the *fraB* mutant does not. Surprisingly, we found that the *fraB* mutant does not grow when both F-Asn and glucose are provided together (Fig. 2). We found that the lack of growth on the dual carbon source medium is specific to the *fraB* mutant. Mutants lacking *fraD*, *fraE*, *fraA*, or the entire *fra* island, grow as well as the wild-type in medium containing both F-Asn and glucose (Fig. 2). Because a *fraD* mutation does not have this phenotype, and it encodes the enzyme just upstream of FraB in the pathway, we hypothesized that the putative metabolic intermediate, 6-phosphofructose-aspartate (6-P-F-Asp), is toxic to the cell (Fig. 2). Deletion of both *fraB* and *fraD* simultaneously or *fraB, fraD*, and *fraA*, relieved the toxicity, consistent with the need for FraD to produce the toxic intermediate (Fig. 2).

### 6-P-F-Asp accumulates in the *fraB* mutant

To test the idea that F-Asn toxicity results from the presence of FraD and absence of FraB, we adopted an approach that combined enzymatic assays and mass spectrometry. We first used biochemical assays to measure FraD and FraB activities in crude lysates prepared from wild-type *Salmonella* and in mutants lacking either *fraB, fraD* or the entire *fra* island, all grown for a short duration in 5 mM F-Asn (see *Methods*). These measurements were made possible due to in-house synthesis of F-Asp and 6-P-F-Asp, the substrates of FraD and FraB, respectively^17^, and design of coupled assays that enabled facile colorimetric readouts (Sengupta and Gopalan, manuscript in preparation). Comparing the wild type and *fra* island deletion mutant offers a positive and negative control cohort (Table 1). In the Δ*fraB*::kan mutant, there is near-wild-type FraD but no FraB activity (Table 1). In the *fraD* mutant, there is no FraD activity, and the FraB activity is two-thirds of that observed in the wild type (Table 1); it is possible that deletion of *fraD* led to some modest regulatory changes that dampened FraB expression. Regardless, the overall trends are as expected.

**Figure 1:**
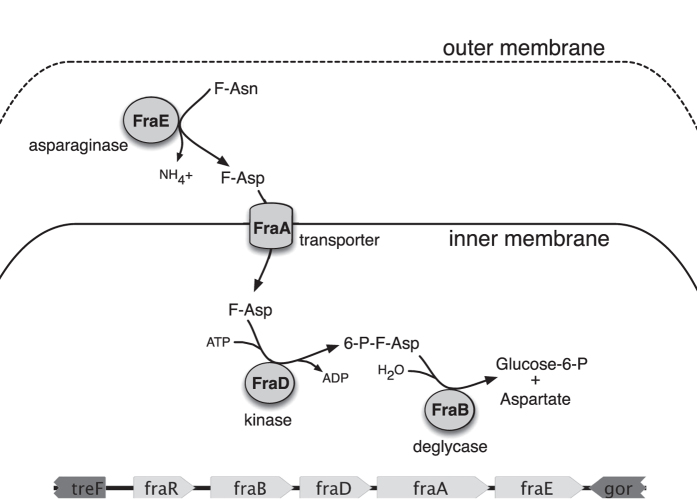
Proposed pathway for fructose-asparagine (F-Asn) utilization in *Salmonella enterica* and genetic organization of the *fra* island. The five *fra* genes are unique to *Salmonella* while the surrounding *treF* and *gor* genes are conserved between *E. coli* and *Salmonella*. A proteomic survey of subcellular fractions determined that FraE is periplasmic while FraB is cytoplasmic^33^. The functions of FraD and FraB were proposed based on homology to the *E. coli* FrlD and FrlB proteins involved in fructose-lysine metabolism^18^, and the inability of a *fraB* mutant to grow on F-Asn^16^. In this report, we confirm that 6-phosphofructose-aspartate (6-P-F-Asp) is the product of FraD and the substrate of FraB.

We next employed mass spectrometry (MS) to measure 6-P-F-Asp levels in the wild type and *fra* mutants. An aliquot of the cells used for the enzyme assays (described above) were used for the MS measurements. 6-P-F-Asp was detected in the *fraB* mutant, but was not detected in the wild-type or the *fra* island deletion mutant (Fig. 3, Supplementary Figs 1–3, Supplementary Tables 1–4). Collectively, results from the biochemical assays and mass spectrometry studies establish that the product generated by FraD is 6-P-F-Asp, which accumulates in the absence of FraB, as would be expected from the predicted pathway for F-Asn utilization (Fig. 1).

### F-Asn is bacteriostatic, not bactericidal

To determine if F-Asn is bacteriostatic or bactericidal to the *fraB* mutant, we determined the 50% and 90% inhibitory concentration (IC_50_ and IC_90_) and the minimum bactericidal concentration (MBC). The IC_50_ and IC_90_ were determined by adding increasing concentrations of F-Asn to M9 minimal medium containing glucose as the carbon source. Growth of the *fraB* mutant was then monitored in these media. F-Asn did not affect the wild-type or *fra* island mutant but was inhibitory to growth of the *fra*B mutant with an IC_50_ of 19 μM (95% confidence interval of 9 to 40 μM) and IC_90_ of 174 μM (95% confidence interval of 82 to 368 μM) (Fig. 4). To determine the MBC, we removed a small aliquot of these same cultures and performed dilution plating onto LB agar. Such an approach helps determine if the bacteria can recover from the inhibition and grow on a non-inhibitory medium. Since *Salmonella* could recover even at the highest concentrations of F-Asn tested, we could not obtain an MBC. Moreover, these results indicate that the inhibition experienced by a *fraB* mutant grown on F-Asn is bacteriostatic and not bactericidal.

### The phenotype of a *fraB* mutant in mice is due to the toxicity of F-Asn

**Figure 2:**
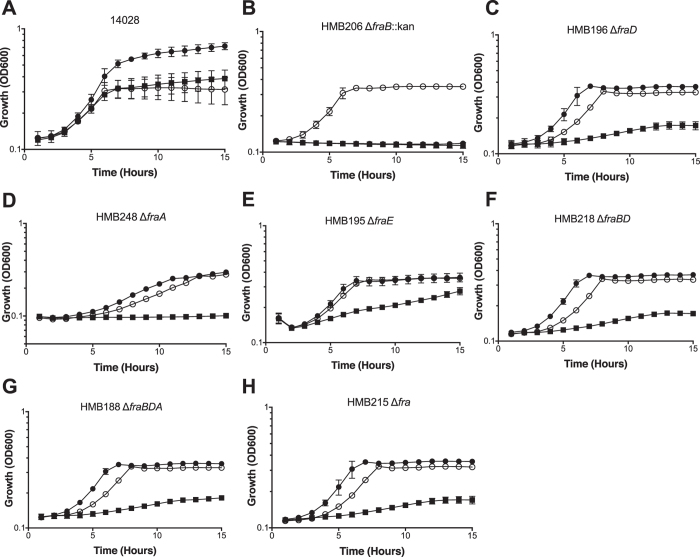
Growth of *fra* mutants on different carbon sources. Wild-type *Salmonella* (14028) and isogenic *fra* mutants were grown in M9 minimal media with ~19 mM ammonium chloride supplemented with either 5 mM glucose (open circles), 5 mM F-Asn (closed squares), or 5 mM glucose and 5 mM F-Asn (closed circles). All data points are the mean of three biological replicates measured in triplicate (9 total points). Error bars represent standard deviation.

We previously determined that a *fraB* mutant had a severe fitness defect (up to 100,000-fold) using competition assays in several mouse models of inflammation, and interpreted this result as a reflection of the importance of F-Asn as a critical nutrient for *Salmonella* in the inflamed intestine^16^. The realization that a *fraB* mutant is inhibited by low concentrations of F-Asn (Fig. 2) provides an alternative explanation based on toxicity of 6-P-F-Asp for the *fraB* fitness defect in mice. To distinguish between these two mechanisms, we tested a *fraB* mutant, a *fraD* mutant, and a mutant lacking the entire *fra* island for fitness in streptomycin-treated Swiss Webster mice (Fig. 5). The expectation was that the *fraD* and *fra* island deletion mutants, which are not inhibited by F-Asn (Fig. 2), should help assess the importance of F-Asn as a nutrient. Neither the *fraD* nor the *fra* island mutant were attenuated in these mice, while the *fraB* mutant was attenuated about 1,000-fold (Fig. 5). Therefore, at least in this streptomycin-treated Swiss Webster mouse model, the entire phenotype of the *fraB* mutant appears to be due to the toxicity of F-Asn rather than an inability to use F-Asn as a nutrient. The decline of the *fraB* mutant CFU may suggest that F-Asn is bactericidal rather than bacteriostatic to the mutant *in vivo* (Fig. 5B). Further studies will be required to test this hypothesis.

## Discussion

This study provides experimental evidence to support a F-Asn catabolic pathway (Fig. 1), which we recently proposed based on the genes in the *fra* locus and on a knowledge of the enzymes required for utilization of F-Lys, another Amadori compound^16^,^18^. Validation of metabolic pathways has typically entailed either assessing the growth of genetic mutants under specific limiting conditions or using biochemical and genetic approaches to cause roadblocks at specific stages in a multi-step conversion to identify the metabolite that accumulates. Here, we have employed both strategies.

**Figure 3:**
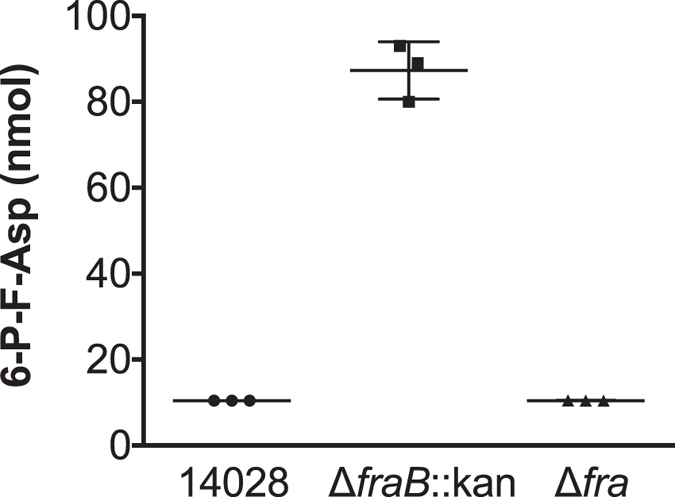
Each point represents mass spectrometry (MS)-based measurement of the levels of 6-P-F-Asp in one biological replicate. Samples were prepared using the cell pellet from a 1.3-ml culture of *Salmonella* wild-type (14028) or Δ*fraB*::kan (HMB206) or Δ*fra* island deletion (HMB215) mutant. Two transitions were measured to confirm quantitation; data from one transition (Table S4B) are shown here, while data from a second transition (Table S4A) are shown in Fig. S3. The values provided are the mean ± standard deviation from three biological replicates. The values for wild-type and Δ*fra* island deletion are very low and below 20 nmol, the lowest concentration used for establishing the standard curve; however, they are provided here to indicate the reproducible absence of 6-P-F-Asp in these two strains.

Mutations in *fraB*, *fraD*, and *fraA* eliminate the ability of *Salmonella* to grow on F-Asn, while a mutation in *fraE* reduces growth on F-Asn (Fig. 2). The residual growth of the *fraE* mutant on F-Asn is likely due to redundancy from other periplasmic asparaginases, a hypothesis that we are testing. Unlike all of the other *fra* mutants, the *fraB* mutant has the unusual property of not being able to grow on glucose if F-Asn is also present (Fig. 2). Since FraB is the terminal enzyme in the pathway, we hypothesized that the substrate of FraB may be toxic to the cell. Through the use of mass spectrometry and *fraB*/*fraD* mutants, we have confirmed that the FraB substrate is 6-P-F-Asp and that 6-P-F-Asp accumulates in a *fraB* mutant (Figs 1 and 3). Moreover, the presence of FraB deglycase and FraD kinase activities in wild-type *Salmonella* crude extracts confirms the postulated route for F-Asn metabolism; the complete absence of FraD and FraB activities in the *fra* island mutant suggests little functional redundancy with respect to F-Asn utilization (Table 1). Ongoing studies with recombinant Fra enzymes are expected to provide additional insights into the individual biochemical transformations in the F-Asn pathway.

**Figure 4:**
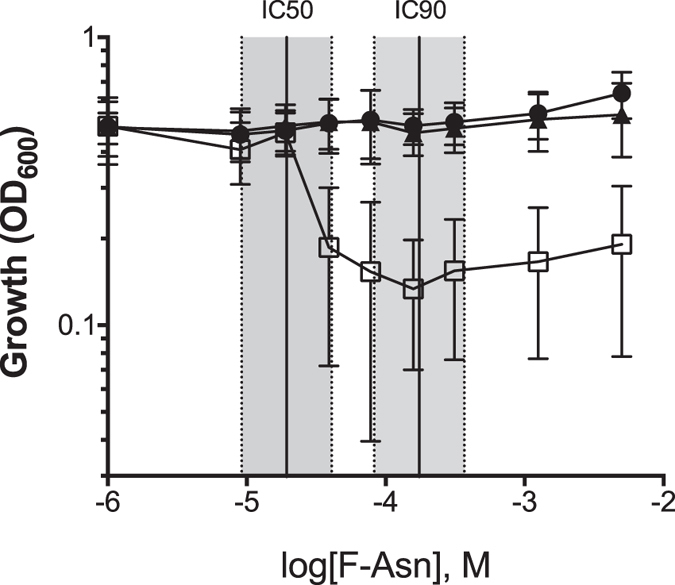
Determination of the IC_50_ and IC_90_ of F-Asn to a *fraB* mutant. Wild-type (14028, closed circles), Δ*fraB*::kan (HMB206, open squares) or Δ*fra* island (HMB205, closed triangles) were grown in M9 minimal medium containing 5 mM glucose and varying concentrations of F-Asn. The data points represent the optical density obtained after 15 h of growth at 37 °C (three biological replicates with three technical replicates in each (nine total replicates); error bars represent standard deviation. IC_50_ and IC_90_ were calculated and plotted ± the 95% confidence intervals. The IC_50_ is 19 μM (9 to 40 μM) and the IC_90_ is 174 μM (82 to 368 μM).

**Figure 5:**
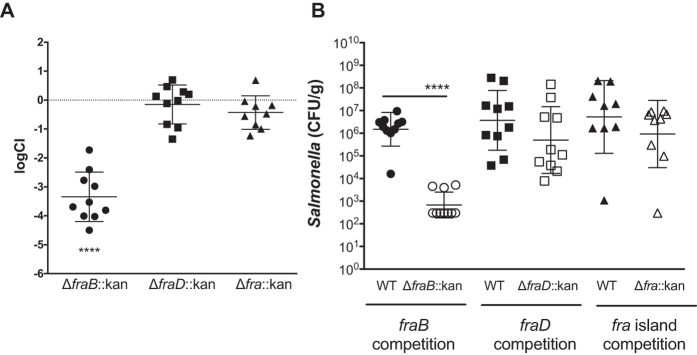
(**A**) Competitive index (CI) of fra mutants *in vivo*. Mixtures (1:1) of wild-type JLD1214 and a mutant (either HMB206 Δ*fraB80*::kan, HMB184 Δ*fraD4*::kan, or HMB205 Δ*fra80*::kan) were administered intragastrically (i.g.) to mice that had been treated 24 h earlier with 20 mg of streptomycin. Mice were monitored for survival and four days post-infection ceca were harvested and plated for CFU. Each point is the CI from one mouse with the geometric mean and standard deviation shown. Statistical significance for each group being different than 0 was calculated using a one-sample, two-tailed t-test (****p-value < 0.0001). (**B**) The raw CFU counts that gave rise to the CI were log transformed and plotted (limit of detection of 300 CFU). Error bars show the mean and standard deviation (****P < 0.0001).

In addition to furthering our understanding of the F-Asn utilization pathway, results from this study revealed an unexpected possibility for inhibiting *Salmonella* growth with clear implications for future drug discovery. Mice with intact microbiota are highly resistant to *Salmonella*-mediated inflammation in the gastrointestinal tract. However, disruption of the microbiota by agents such as streptomycin causes the mice to be susceptible to *Salmonella*-mediated inflammation. Similarly, germ-free mice and *IL10*-deficient mice are also susceptible. We previously reported that two independently constructed *fraB*::kan mutants of *Salmonella* were dramatically attenuated in all of the mouse models that are susceptible to inflammation but not in conventional mice^16^. Because both mutants could be complemented with a plasmid encoding the *fra* island^16^, we inferred that F-Asn is of utmost importance to *Salmonella* as a nutrient during growth in the inflamed intestine. However, we have now discovered that the phenotype is not due to the importance of F-Asn as a nutrient but is rather due to the accumulation of an inhibitory metabolite in the F-Asn utilization pathway: 6-P-F-Asp. Several lines of evidence support this claim. First, observations from growth of different *fra* mutants in minimal medium containing both glucose and F-Asn as carbon sources proved instructive. A mutant lacking *fraD*, or the entire *fra* island, can grow in this medium, while a *fraB* mutant cannot (Figs 2 and 4). Second, mass spectrometric studies demonstrate that 6-P-F-Asp accumulates to high levels in the *fraB* mutant, but not in a *fra* island deletion mutant (Fig. 3). Third, a *fraB* mutant is extremely attenuated in streptomycin-treated Swiss Webster mice, while a *fraD* mutant and a mutant lacking the entire *fra* island have no fitness defect (Fig. 5). Since neither a *fraD* mutant nor a mutant lacking the entire *fra* island can grow on F-Asn, their failure to utilize F-Asn in this particular mouse model does not result in a measurable fitness defect and demonstrates that F-Asn is not an essential nutrient during infection. Last, the organization of the *fra* operon suggests a strategy adopted by *Salmonella* to prevent build-up of 6-P-F-Asp - the *fra* genes are encoded in the opposite order of the enzymatic pathway (Fig. 1), possibly to ensure that FraB is expressed before FraD thereby avoiding an accumulation of the FraD product.

Except for *Salmonella*, only a few *Citrobacter* and *Clostridia* seem to have the *fra* genes although there is no experimental evidence for F-Asn utilization by the latter^16^. Thus, FraB represents a potential drug target whose inhibition will selectively affect *Salmonella* and perhaps a few members of the microbiota. As with numerous other genes involved with anaerobic metabolism in the gut, the *fra* locus is widely distributed and conserved among the gastrointestinal serovars of *Salmonella*, but is missing or mutated in serovars of the extraintestinal pathovar (the typhoidal serovars)^1^. Any inhibitor of FraB would only work in the presence of F-Asn. Given the IC_50_ of F-Asn for a a *FraB* mutant in vitro is 19 μM (in M9 minimal medium containing 5 mM glucose), the amount of F-Asn required for toxicity in vivo is likely to be low and easily met in the diet, as evident from the attenuation of a *fraB* mutant in mice^16^. The concentration of F-Asn has only been measured in a few human foods, but F-Asn in some vegetables (e.g., asparagus) is as high as 1.4% of dry weight^19^,^20^. Since the F-Asn concentration in a human with Salmonellosis would not be known, a FraB inhibitor could be administered with F-Asn to ensure the inhibition of *Salmonella*. F-Asn would only be available to *Salmonella* during inflammation. When inflammation is relieved, the microbiota would be restored, and F-Asn levels would decrease. It is probable that loss-of-function mutations within *fraD* or *fraA* would provide resistance to the inhibitor, but selection for these mutants is likely to be brief, and cease when symptoms cease.

Sugar phosphates (e.g., those of rhamnose, glucose, arabinose, and galactose) are known to be toxic to *E. coli* and *Salmonella*, in part due to depletion of glycolytic metabolites or biosynthetic precursors^21^,^22^,^23^,^24^,^25^,^26^. While we do not know the basis for 6-P-F-Asp toxicity, the F-Asn catabolic pathway has two major advantages over these other pathways with regard to drug discovery. First, the F-Asn pathway is more specific to *Salmonella*, so fewer members of the microbiota are likely to be adversely affected by an inhibitor. Second, the toxicity of the glucose and galactose catabolic pathways can be overcome by the addition of other nutrients *in vitro*^21^,^22^,^27^,^28^. The observation that only the F-Asn utilization pathway was identified in our transposon site hybridization screening in mice, is consistent with the idea that F-Asn toxicity cannot be overcome by the presence of other nutrients that are available in the inflamed intestine^16^. It is also possible that a FraB inhibitor would be bactericidal to *Salmonella in vivo* rather than bacteriostatic, as reflected by the precipitous decline in the CFU of the *fraB* mutant from mice (Fig. 5B). Additional experiments are required to test this hypothesis, and the possibility that additional stressors in the inflamed intestine, that are not present in our in vitro assays, combine with 6-PF-Asp toxicity to kill Salmonella *in vivo*.

## Methods

### Strains and Media

Strains used in this study are listed in Table 2. Bacteria were routinely grown in Luria-Bertani (LB) broth (EMB) or on LB agar plates made by adding 1.5% (w/v) agar (Fisher Bioreagents). For growth studies involving F-Asn, we employed M9 minimal medium^1^,^29^: 1× M9 salts, 2 mM MgSO_4_, 0.1 mM CaCl_2_, 0.01 mM thiamine, and trace metals^1^,^30^. For the growth assays using minimal media without nitrogen, NH_4_Cl was not included in the 10× M9 media. As needed, chloramphenicol (cam, 10 μg/mL) or kanamycin (kan, 50 μg/mL) were added to the media.

### Construction of mutants

Lambda Red mutagenesis was used to generate insertion mutations or in-frame deletions of target genes^10^,^31^. Oligonucleotides containing 40 nucleotides of identity to the target genes were appended to sequences that bind the P1 and P2 sites of pKD4 or pKD3^11^,^31^. Primer sequences used are listed in Table 3. The FRT antibiotic resistance cassette of either pKD3 or pKD4 was amplified by PCR to generate a product with 40 bp identity to target genes on each end. This PCR product was electroporated into strain 14028 + pKD46, and homologous recombinants were selected using LB kan at 37 °C. Correct insertion of the FRT antibiotic cassette was confirmed by PCR using one primer within the antibiotic cassette and another outside the region of homologous recombination. The *bona fide* mutants were transduced into 14028 using phage P22HTint. The antibiotic cassette was then removed by electroporating pCP20 (amp^r^), which encodes FLP recombinase, into the strain and plating on LB amp at 30 °C. Single colonies were streaked onto LB and incubated at 42 °C to cure the strain of pCP20. PCR was used to verify loss of the antibiotic resistance cassette using primers upstream and downstream of the target gene. Colonies with the correct PCR product were also screened for loss of the antibiotic resistance gene and pCP20 (amp^r^).

### Growth assays

Growth curves were performed using clear, flat-bottom, 96-well plates. Minimal media with the specified carbon source was prepared and overnight cultures were washed twice with sterile water. In each well, 198-μl aliquots of media were inoculated with 2 μl of washed overnight cultures. A Breathe-Easy membrane film (Diversified Biotech) was placed over the 96-well plate. Growth over 18 h at 37 °C was measured using hourly OD_600_ measurements in the SpectraMax M5 (Molecular Devices) Microplate Reader and the SoftMax Pro 6.1 software.

### Synthesis of fructose-asparagine and 6-phosphofructose-aspartate

We have previously synthesized fructose-asparagine^16^ with the reaction carried out in refluxing methanol. However, the yields were poor due to the low solubility of asparagine in methanol. Prof. Valeri Mossine (University of Missouri) suggested an approach to overcome this solubility problem: dissolve the components in a small amount of water, add glycerol (or ethylene glycol), and finally remove the water by rotary evaporation. Remarkably, with this method, all of the components remain in solution. Glucose (1 g, 5.5 mmol) and potassium L-aspartate (0.2 g, 1.2 mmol) were dissolved in 3 mL water. Glycerol (3 g) was added and the mixture rotary evaporated at 50 °C to remove the water. The viscous homogeneous solution was heated at 60 °C in an unstoppered flask for 36 h. Glycerol was removed by dissolution in isopropanol. The precipitated product was dissolved in water and applied to a Dowex-50 column in the hydrogen ion form as reported for the synthesis of F-Asn^16^. Mass spectrometry in the negative ion mode showed [fructose aspartic acid]^−^ at m/z 294 (100%) and [aspartic acid]^−^ at m/z 132 (40%). 6-Phosphofructose-aspartate (6-P-F-Asp) could be made only by the glycerol (or ethylene glycol) procedure because the starting material, glucose-6-phosphate, is insoluble in methanol. 6-P-F-Asp was characterized by mass spectrometry and by both proton and ^13^C NMR, and these details are provided elsewhere^17^.

### Preparation of *Salmonella* extracts

*Salmonella* were grown in 20 ml LB for 16 h at 37 °C with shaking. The cells were harvested by centrifugation, re-suspended in 20 ml fresh LB supplemented with 5 mM F-Asn, and grown for 30 min at 37 °C with shaking. These cells were subjected to two cycles of centrifugation (5,000 g at 4 °C) and washes with water, before re-suspension in 1 ml of 25 mM HEPES (pH 7.5), 0.1 mM phenylmethylsulfonyl fluoride. Cells were then lysed by sonication (50% output power for 60 s, with cycles of 2 s on and 5 s off; Ultrasonic Processor, Cole-Parmer), and debris removed by centrifuging the cell lysate at 13,000 g for 20 min at 4 °C. At this point the samples were split with half for enzymatic assays and half for mass spectrometry measurements. For enzymatic assays, after addition of 0.1 mg/ml BSA, the supernatant was dialyzed against 25 mM HEPES (pH 7.5) at 4 °C with two changes over 60 min. The crude dialysates were used for the activity assays described below. To calculate specific activities, the protein content in the crude dialysates was determined using the Bradford assay^32^, with bovine serum albumin serving as the standard.

### Enzyme assays

All assays (40 μl volume) were carried out at 37 °C. For the measurement of FraB deglycase activity, we used a glucose-6-phosphate dehydrogenase (G6PD)-based coupled assay. The FraB reaction mixture contained 1 mM 6-P-F-Asp, 25 mM HEPES (pH 7.5), 5 mM MgCl_2_, 0.1 mM EGTA, 0.5 mM NADP^+^, 0.15 U G6PD (Sigma, G6378). The reaction was initiated by the addition of a defined amount of crude lysates (up to 40% of the assay volume) obtained from wild-type or mutant *Salmonella* strains. The NADPH generated by G6PD was followed by measuring absorbance at 340 nm and taken as a direct readout of the glucose-6-phosphate produced by FraB. To determine FraD kinase activity, a G6PDH + FraB-based coupled assay was performed. The reaction mixture for the kinase assay contained 1 mM fructose-aspartate (F-Asp), 25 mM HEPES (pH 7.5), 25 mM KCl, 1 mM MgCl_2_, 1 mM dithiothreitol, 1 mM ATP, 0.1 mM EGTA, 0.5 mM NADP^+^, 0.3 μM recombinant FraB (Sengupta and Gopalan, unpublished) and 0.15 U G6PD (Sigma, G6378). For both FraB and FraD assays, the reactions were terminated by addition of 6 mM EDTA (final concentration). One unit of activity is defined as the amount of enzyme catalyzing the formation of 1 μmol of NADPH per min. Mean and standard deviation values were calculated from independent assays that used crude lysates from three separate cultures.

### Mass spectrometry

To measure intracellular 6-P-F-Asp, the bacteria were grown as described above for preparation of *Salmonella* extracts and the cell pellets were re-suspended in 20 ml water. The cell suspension was divided into 15 aliquots and one aliquot was used for each of the following analyses/replicates. Cells were lysed by two cycles of freeze thaw (30 s at −20 °C, followed by 90 s at 37 °C), followed by 120 s of sonication. Six hundred μl of chilled methanol (Fisher Optima grade, Fisher Scientific) with 16 nmol [^13^C]-F-Asn (internal standard) was added. Five wild-type Salmonella aliquots were spiked with 0, 20, 40, 160 or 320 nmol of 6-P-F-Asp to generate a standard curve. The cell suspension was then vortexed and incubated on ice for 30 min, followed by the addition of 600 μl of dichloromethane (Sigma-Aldrich). After being vortexed and centrifuged at 16,200 g for 10 min at 4 °C, the upper layer (aqueous phase) was carefully transferred into a new tube with little disturbance of the rest of the mixture. Six hundred μl of chilled acetonitrile (Fisher Optima LC/MS grade, Fisher Scientific) was added, followed by vortexing and incubation at −80 °C for 2 h. These samples were then centrifuged at 16,200 g for 20 min at 4 °C. The supernatants were transferred to new tubes and dried under vacuum (SpeedVac Concentrator, Thermo Scientific). Before mass spectrometry analysis, these dried pellets were resuspended in 900 μl methanol/water, 50%:50% with 0.1% (v/v) formic acid (LC-MS grade, Thermo Scientific). Samples were introduced into a triple quadrupole mass spectrometer (Waters Xevo TQ-S) by direct infusion at a flow rate of 7 μl/min. The mass spectrometer was operated in positive ion electrospray ionization mode (ESI+) with capillary voltage 3 kV, source temperature 150 °C, cone voltage 2 V, cone flow 150 l/h, source offset 2 V, desolvation temperature 350 °C, desolvation gas flow 350 l/h and nebulizer gas flow 7 bar. The gas flow rate for the collision cell was 0.15 ml/min. While transitions m/z 376 → 125 and m/z 376 → 242 of 6-P-F-Asp with collision energy 15 eV were selected for quantitation, m/z 301 → 216 of [_13_C]-F-Asn with collision energy 13 eV was used for normalization. Acquired data were analyzed using Masslynx 4.1. A total of 22 MS/MS scans of each ion were averaged for the measurement of ion intensity.

### Competition experiments

A mutant strain, either *fraB*::kan HMB206, *fraD*::kan HMB184 or *fra80*::kan HMB205, were mixed with the isogenic wild-type strain (JLD1214), respectively, and inoculated by the intragastric (i.g.) route to Swiss Webster mice treated with 20 mg of streptomycin 24 h prior. Ceca samples were collected five days post-inoculation, homogenized, and plated on XLD kan and XLD cam plates. Antibiotic resistance differentiated the mutant and wild-type strains. The competitive index was calculated as CI = (cfu mutant recovered/cfu wild-type recovered)/(cfu mutant input/cfu wild-type input).

### Animal assurance

All animal work was performed using protocols approved by our Institutional Animal Care and Use Committee (IACUC; OSU 2009A0035) and in accordance with the relevant guidelines set forth in the PHS “Guide for the Care and Use of Laboratory Animals”.

## Additional Information

**How to cite this article**: Sabag-Daigle, A. *et al*. A metabolic intermediate of the fructose-asparagine utilization pathway inhibits growth of a *Salmonella fraB* mutant. *Sci. Rep.* **6**, 28117; doi: 10.1038/srep28117 (2016).

## Supplementary Material

Supplementary Information

## Figures and Tables

**Table 1:** Specific activities of FraD (kinase) and FraB (deglycase) in *Salmonella* wild-type and mutant extracts.

	FraD (×10^4^ U/mg)	FraB (×10^4^ U/mg)
Wild-type	2.4 ± 0.13	1.3 ± 0.16
*∆fraB::*kan	2.2 ± 0.15	ND
*∆fraD*	ND	0.9 ± 0.06
*∆fra*	ND	ND

The specific activities reported are the mean and standard deviation values from three independent experiments (see *Methods*); ND - not detectable.

**Table 2:** Strains and Plasmids.

Strains	Genotype	Reference or construction
14028	Wild-type *Salmonella enterica* subspecies *enterica* serovar Typhimurium	ATCC
HMB176	14028 Δ*fraBDA80*::cam	lambda red mutation of *fraBDA* made using PCR primers BA2553 and BA2511 and transduced into a clean 14028 background.
HMB182	14028 Δ*fraE4*::kan	lambda red mutation of *fraE* made using PCR primers BA2537 and BA2515 and transduced into a clean 14028 background.
HMB184	14028 Δ*fraD4*::kan	lambda red mutation of *fraD* made using PCR primers BA2494 and BA2495 and transduced into a clean 14028 background.
HMB188	14028 Δ*fraBDA80*	Antibiotic cassette in HMB176 was flipped out using pCP20.
HMB195	14028 Δ*fraE4*	Antibiotic cassette in HMB182 was flipped out using pCP20.
HMB196	14028 Δ*fraD4*	Antibiotic cassette in HMB184 was flipped out using pCP20.
HMB205	14028 Δ*fra80*::kan	lambda red mutation of *fra* island made using PCR primers BA2538 and BA2513 and transduced into a clean 14028 background.
HMB206	14028 Δ*fraB80*::kan	lambda red mutation of *fraB* made using PCR primers BA2552 and BA2553 and transduced into a clean 14028 background.
HMB211	14028 Δ*fraBD81*::kan	lambda red mutation of *fraBD* made using PCR primers BA2553 and BA2495 and transduced into a clean 14028 background.
HMB215	14028 Δ*fra80*	Antibiotic cassette in HMB205 was flipped out using pCP20.
HMB218	14028 Δ*fraBD81*	Antibiotic cassette in HMB211 was flipped out using pCP20.
HMB247	14028 Δ*fraA4*::kan	lambda red mutation of *fraA* made using PCR primers BA2510 and BA2511 and transduced into a clean 14028 background.
HMB248	14028 Δ*fraA4*	Antibiotic cassette in HMB247 was flipped out using pCP20.
JLD1214	14028 IG (*pagC*-STM14_1502)::cam	16
**Plasmids**	**Description**	**Reference**
pKD46	P_BAD_ *gam bet exo* pSC101 *oriTS* (amp^r^)	31
pKD3	FRT-*cam*-FRT *oriR6K* (amp^r^)	31
pKD4	FRT-*kan*-FRT *oriR6K* (amp^r^)	31
pCP20	cI857 λPR *flp* pSC101 *oriTS* (amp^r^ cam^r^)	34

**Table 3:** Oligonucleotides.

Primer	Sequence	Description
BA2494	ATTGTAAAGACAAACAAGGAATAATGATGATGTGTAGGCTGGAGCTGCTTC	forward primer for *fraD* lambda red mutagenesis
BA2495	TACATTGAGGGACGTAACCTATTGTGCAAACATATGAATATCCTCCTTA	reverse primer for *fraD* and *fraBD* lambda red mutagenesis
BA2510	AGGAGGAAGTATGTTTTGGACGGAATTATGTTTTATCCTTGTGTAGGCTGGAGCTGCTTC	forward primer for *fraA* lambda red mutagenesis
BA2511	TGAATAACAATCAGGCCAGAACCATTTTTCCTATTAACAGCATATGAATATCCTCCTTAG	reverse primer for *fraA* and *fraBDA* lambda red mutagenesis
BA2513	GCGCACAAGCCTGCATGATTAATACGTACTCATATGAATATCCTCCTTAG	reverse primer for *fra* island lambda red mutagenesis
BA2515	GCCTGCATGATTAATACGTACTGAAATAACTCTGGATCAGCATATGAATATCCTCCTTAG	reverse primer for *fraE* lambda red mutagenesis
BA2537	GAGGAAGAAAATGAAAATTAGAGTTTTCATGGCCACCGTGGTGTAGGCTGGAGCTGCTTC	forward primer for *fraE* lambda red mutagenesis
BA2538	ATGGATACAAATGATCGAGCAACCCGACAGTAAAAGCGCCGTGTAGGCTGGAGCTGCTTC	forward primer for *fraR* and *fra* island lambda red mutagenesis
BA2539	AATGCTGACATCATACGGTAAACCGTATTTTATCGCCGACCATATGAATATCCTCCTTAG	reverse primer for *fraR* lambda red mutagenesis
BA2552	CCTGATGTAATTAATATTCCACTTTCCACATATAGCGGCGCATATGAATATCCTCCTTAG	forward primer for *fraB* lambda red mutagenesis
BA2553	AGAGGAAAGCATGATGGGTATGAAAGAGACAGTTAGCAATGTGTAGGCTGGAGCTGCTTC	reverse primer for *fraB* and *fra*BDA and *fraBD* lambda red mutagenesis
