# Supplementary material for: A metabolic intermediate of the fructose-asparagine utilization pathway inhibits growth of a Salmonella fraB mutant
Source: Sci Rep. 2016 Jul 12;6:28117. doi: 10.1038/srep28117 (PMC4941530; doi:10.1038/srep28117)
Supplement: Supplementary Information [file srep28117-s1.pdf]

Supplementary information for:

A metabolic intermediate of the fructose-asparagine utilization pathway inhibits growth of a *Salmonella fraB* mutant

Anice Sabag-Daigle[1,2], Henry M. Blunk[3], Anindita Sengupta[4], Jikang Wu[4], Alexander J. Bogard[4], Mohamed M. Ali[2,3,5], Christopher Stahl[3], Vicki H. Wysocki[4], Venkat Gopalan[4], Edward J. Behrman[4], and Brian M. M. Ahmer[1,2,3]*

[1] Department of Microbial Infection and Immunity, [2] Center for Microbial Interface Biology, [3] Department of Microbiology, [4] Department of Chemistry and Biochemistry, The Ohio State University, Columbus, OH 43210.

[5] Department of Medical Microbiology and Immunology, Faculty of Medicine, Mansoura University, Mansoura, Egypt.

* Corresponding Author:

The Ohio State University

Department of Microbial Infection and Immunity.

460 West 12th Ave

710 Biomedical Research Tower

Columbus, OH 43210

614-292-1919

ahmer.1@osu.edu

Supplementary Table S1. The intensity of transitions at 22 different run times and the average of the intensity from 22 scans (in bold) from *Salmonella* wild-type or Δ*fraB*::kan or Δ*fra* island. S1A: Transition m/z 376→242 of 6-P-F-Asp with collision energy 15 eV. S1B: Transition m/z 376→125 of 6-P-F-Asp with collision energy 15 eV. S1C: Transition m/z 301→216 of spiked internal standard [^{13}C]-F-Asn with collision energy 13 eV.

**Supplementary Table S1A**			**Transition 376→125**			
	Run Time (min)	Intensity	Run Time (min)	Intensity	Run Time (min)	Intensity
wild-type	Replicate 1		Replicate 2		Replicate 3	
	0.00	0.00	0.00	229.00	0.00	0.00
	0.09	0.00	0.09	114.48	0.09	0.00
	0.17	0.00	0.17	0.00	0.17	0.00
	0.26	0.00	0.26	0.00	0.26	0.00
	0.34	0.00	0.34	0.00	0.34	0.00
	0.43	0.00	0.43	0.00	0.43	0.07
	0.51	0.00	0.51	0.00	0.51	89.00
	0.60	0.96	0.60	0.00	0.60	84.90
	0.69	326.63	0.69	0.00	0.69	0.00
	0.77	207.72	0.77	0.00	0.77	0.00
	0.86	89.77	0.86	0.00	0.86	0.00
	0.94	0.00	0.94	0.00	0.94	0.00
	1.03	0.00	1.03	0.00	1.03	0.00
	1.11	0.00	1.11	0.00	1.11	0.00
	1.20	0.00	1.20	0.00	1.20	0.00
	1.28	0.00	1.28	0.00	1.28	0.00
	1.37	0.00	1.37	0.00	1.37	0.00
	1.45	0.00	1.45	0.00	1.45	0.00
	1.54	0.33	1.54	0.00	1.54	0.00
	1.62	89.98	1.62	0.00	1.62	0.00
	1.71	85.66	1.71	0.00	1.71	0.00
	1.80	0.00	1.80	0.00	1.80	0.00
**Average from 22 scans**		**36.41**		**15.61**		**7.91**
Δ*fraB*::kan	Replicate 1		Replicate 2		Replicate 3	
	0.00	105296.00	0.00	65520.00	0.00	89232.00
	0.09	83696.00	0.09	84603.73	0.09	82414.67
	0.17	65230.20	0.17	103671.09	0.17	75604.77
	0.26	46777.56	0.26	80855.27	0.26	87835.94
	0.34	95770.02	0.34	118093.44	0.34	108228.19
	0.43	65152.00	0.43	70618.93	0.43	113559.35
	0.51	95792.00	0.51	105109.55	0.51	88382.99
	0.60	12168.00	0.60	132641.92	0.60	122746.23
	0.69	57156.78	0.69	101877.70	0.69	140669.59
	0.77	102122.12	0.77	120417.38	0.77	112332.52
	0.86	87095.04	0.86	135178.14	0.86	92192.20

	0.94	99109.91	0.94	95788.18	0.94	127183.06
	1.03	109205.92	1.03	92546.17	1.03	88713.41
	1.11	119287.68	1.11	105802.41	1.11	86768.27
	1.20	108630.23	1.20	92026.16	1.20	134746.94
	1.28	98615.30	1.28	111126.88	1.28	85240.96
	1.37	91205.70	1.37	105286.88	1.37	100656.62
	1.45	94853.72	1.45	99465.04	1.45	129253.20
	1.54	98500.62	1.54	105218.18	1.54	95524.79
	1.62	90189.70	1.62	89972.77	1.62	93066.41
	1.71	98889.53	1.71	92224.35	1.71	97792.73
	1.80	67980.44	1.80	106004.67	1.80	84411.25
**Average from 22 scans**		**86032.93**		**100638.58**		**101661.64**
Δ*fra* island	Replicate 1		Replicate 2		Replicate 3	
	0.00	88.00	0.00	330.00	0.00	298.00
	0.09	43.99	0.09	164.97	0.09	148.99
	0.17	0.00	0.17	0.04	0.17	0.00
	0.26	0.00	0.26	89.95	0.26	0.00
	0.34	0.00	0.34	0.00	0.34	0.00
	0.43	0.00	0.43	0.00	0.43	0.00
	0.51	0.00	0.51	0.00	0.51	0.04
	0.60	0.00	0.60	0.00	0.60	92.00
	0.69	0.00	0.69	0.00	0.69	89.00
	0.77	0.00	0.77	0.00	0.77	91.82
	0.86	0.00	0.86	0.00	0.86	0.00
	0.94	0.00	0.94	0.00	0.94	0.00
	1.03	0.00	1.03	0.00	1.03	0.00
	1.11	0.00	1.11	0.00	1.11	0.00
	1.20	0.00	1.20	0.00	1.20	0.00
	1.28	0.00	1.28	0.00	1.28	0.00
	1.37	0.00	1.37	0.00	1.37	0.00
	1.45	0.00	1.45	0.18	1.45	0.00
	1.54	0.00	1.54	90.00	1.54	0.00
	1.62	0.00	1.62	89.77	1.62	0.00
	1.71	0.00	1.71	0.00	1.71	0.00
	1.80	0.00	1.80	0.00	1.80	0.00
**Average from 22 scans**		**6.00**		**34.77**		**32.72**

Supplementary Table S1B			Transition 376→242			
	Run Time (min)	Intensity	Run Time (min)	Intensity	Run Time (min)	Intensity
wild-type	Replicate 1		Replicate 2		Replicate 3	
	0.00	0.00	0.00	109.00	0.00	0.00
	0.09	0.00	0.09	54.49	0.09	49.50
	0.17	0.00	0.17	0.00	0.17	99.00
	0.26	0.00	0.26	0.00	0.26	49.49
	0.34	0.00	0.34	0.00	0.34	0.00
	0.43	0.00	0.43	0.00	0.43	0.00
	0.51	0.00	0.51	0.00	0.51	0.00
	0.60	0.00	0.60	0.00	0.60	0.00
	0.69	0.00	0.69	0.00	0.69	0.00
	0.77	0.00	0.77	0.00	0.77	0.38
	0.86	0.00	0.86	0.00	0.86	278.78
	0.94	0.00	0.94	0.00	0.94	138.76
	1.03	0.00	1.03	0.00	1.03	0.00
	1.11	0.00	1.11	0.00	1.11	0.00
	1.20	0.00	1.20	0.00	1.20	0.00
	1.28	0.00	1.28	0.00	1.28	0.00
	1.37	0.00	1.37	0.00	1.37	0.00
	1.45	0.00	1.45	0.00	1.45	0.00
	1.54	0.00	1.54	0.00	1.54	0.00
	1.62	0.00	1.62	0.00	1.62	0.00
	1.71	0.00	1.71	0.00	1.71	0.00
	1.80	0.00	1.80	0.00	1.80	0.00
**Average from 22 scans**		**0.00**		**7.43**		**28.00**
Δ*fraB*::kan	Replicate 1		Replicate 2		Replicate 3	
	0.00	378432.00	0.00	428864.00	0.00	412224.00
	0.09	434240.00	0.09	374805.47	0.09	347475.38
	0.17	408061.44	0.17	320806.84	0.17	282805.72
	0.26	381898.41	0.26	420285.44	0.26	420268.75
	0.34	435263.19	0.34	407189.94	0.34	278215.00
	0.43	431104.00	0.43	463278.56	0.43	369078.25
	0.51	478208.00	0.51	440894.00	0.51	359033.22
	0.60	274688.00	0.60	356460.31	0.60	307317.38
	0.69	297284.41	0.69	336371.69	0.69	302773.34
	0.77	319870.31	0.77	380480.91	0.77	298595.00
	0.86	315561.25	0.86	381248.97	0.86	438069.50
	0.94	386010.47	0.94	381960.84	0.94	410904.91
	1.03	431163.59	1.03	389514.41	1.03	419113.97
	1.11	423654.38	1.11	397131.19	1.11	330717.13
	1.20	390840.69	1.20	404285.72	1.20	435400.47
	1.28	383351.31	1.28	402621.41	1.28	380231.28
	1.37	375837.22	1.37	400938.03	1.37	365068.44
	1.45	312673.84	1.45	386895.50	1.45	384534.28
	1.54	334365.72	1.54	396819.50	1.54	374184.94

	1.62	356220.00	1.62	406959.50	1.62	326823.66
	1.71	435390.53	1.71	488512.44	1.71	380461.09
	1.80	398142.69	1.80	488759.53	1.80	355754.28
**Average from 22 scans**		**381011.88**		**402503.83**		**362684.09**
Δ*fra* island	Replicate 1		Replicate 2		Replicate 3	
	0.00	92.00	0.00	0.00	0.00	0.00
	0.09	1564.29	0.09	0.00	0.09	0.00
	0.17	3034.81	0.17	0.00	0.17	0.00
	0.26	0.00	0.26	0.00	0.26	0.00
	0.34	0.00	0.34	0.00	0.34	0.00
	0.43	0.00	0.43	0.00	0.43	0.00
	0.51	0.00	0.51	0.00	0.51	0.00
	0.60	0.00	0.60	0.00	0.60	0.00
	0.69	0.00	0.69	1.69	0.69	0.00
	0.77	0.00	0.77	959.06	0.77	0.08
	0.86	0.00	0.86	479.06	0.86	42.96
	0.94	0.00	0.94	0.19	0.94	20.95
	1.03	0.00	1.03	89.84	1.03	0.00
	1.11	0.00	1.11	0.00	1.11	0.00
	1.20	0.00	1.20	0.00	1.20	0.00
	1.28	0.00	1.28	0.00	1.28	0.00
	1.37	0.00	1.37	0.00	1.37	0.00
	1.45	0.00	1.45	0.00	1.45	0.00
	1.54	0.00	1.54	0.00	1.54	0.00
	1.62	0.00	1.62	0.00	1.62	0.00
	1.71	0.00	1.71	0.00	1.71	0.00
	1.80	0.00	1.80	0.00	1.80	0.00

Supplementary Table S1C			Transition 301→216			
	Run Time (min)	Intensity	Run Time (min)	Intensity	Run Time (min)	Intensity
wild-type	Replicate 1		Replicate 2		Replicate 3	
	0.00	4855808.00	0.00	5217280.00	0.00	6034432.00
	0.09	4628947.50	0.09	5341720.50	0.09	6182912.00
	0.17	4402241.00	0.17	5466081.00	0.17	6331392.00
	0.26	4569217.50	0.26	5386110.00	0.26	5697567.50
	0.34	4734894.50	0.34	5290941.00	0.34	5859561.50
	0.43	4665233.50	0.43	5248424.50	0.43	6456441.50
	0.51	4594024.00	0.51	5610678.00	0.51	5301400.00
	0.60	4256597.00	0.60	5743850.00	0.60	5456564.00
	0.68	4893616.00	0.68	5876838.00	0.68	5173426.50
	0.77	4216108.00	0.77	5928141.00	0.77	5325438.00
	0.86	5215172.50	0.86	5508641.50	0.86	5043803.00
	0.94	4789414.50	0.94	5159774.00	0.94	5429924.50
	1.03	4852814.50	1.03	5788823.50	1.03	5816013.00
	1.11	4136416.50	1.11	5231310.00	1.11	6805656.00
	1.20	4996915.50	1.20	5639260.00	1.20	6880494.00
	1.28	4931519.50	1.28	5685614.50	1.28	5431173.00
	1.37	4909915.00	1.37	5380187.00	1.37	5376496.50
	1.45	4283769.00	1.45	5416060.50	1.45	6373634.00
	1.54	4658549.50	1.54	4259582.50	1.54	5346304.00
	1.62	4206973.50	1.62	4915073.50	1.62	5345182.00
	1.71	3782887.25	1.71	5569552.00	1.71	4937542.00
	1.79	4105774.75	1.79	5574673.00	1.79	5573198.50
**Average from 22 scans**		**4576673.14**		**5419937.09**		**5735388.89**
Δ*fraB*::kan	Replicate 1		Replicate 2		Replicate 3	
	0.00	6390784.00	0.00	6366208.00	0.00	4790272.00
	0.09	4556800.00	0.09	6036927.50	0.09	4767739.50
	0.17	6023823.00	0.17	5707516.00	0.17	4745121.50
	0.26	7490504.50	0.26	5041227.50	0.26	4502374.50
	0.34	7205162.50	0.34	5428375.00	0.34	4240588.00
	0.43	3493888.00	0.43	5814381.50	0.43	4502261.50
	0.51	6437888.00	0.51	5954668.50	0.51	5277353.00
	0.60	5686272.00	0.60	6093648.00	0.60	5024914.00
	0.68	5890600.00	0.68	5912835.00	0.68	4216811.00
	0.77	6094809.00	0.77	5260065.00	0.77	5080370.50
	0.86	5995117.00	0.86	5069989.00	0.86	5201254.00
	0.94	5308190.00	0.94	5190807.00	0.94	5331353.50
	1.03	6670302.50	1.03	5345509.50	1.03	5120014.00
	1.11	6613612.50	1.11	5513480.00	1.11	4473590.50
	1.20	7405299.50	1.20	5682643.50	1.20	5316013.00
	1.28	6083772.00	1.28	6023724.50	1.28	5134734.00

	1.37	6967314.00	1.37	5681643.50	1.37	4420241.50
	1.45	5669392.50	1.45	5341507.50	1.45	5369922.50
	1.54	5353365.00	1.54	5527297.00	1.54	4889648.00
	1.62	6022914.00	1.62	6444041.00	1.62	5127464.50
	1.71	5861864.50	1.71	6446893.50	1.71	5181757.50
	1.79	6721140.50	1.79	5921137.00	1.79	5233421.50
**Average from 22 scans**		**6088309.77**		**5718387.50**		**4906691.82**
Δ*fra* island	Replicate 1		Replicate 2		Replicate 3	
	0.00	7135232.00	0.00	6286336.00	0.00	5451776.00
	0.09	6648225.00	0.09	5635457.00	0.09	5424637.50
	0.17	6161864.00	0.17	4984942.50	0.17	5397431.50
	0.26	7329131.00	0.26	5268512.00	0.26	5026779.00
	0.34	6201920.50	0.34	5322773.00	0.34	4931768.50
	0.43	6940060.50	0.43	5376997.00	0.43	5404398.50
	0.51	7361177.00	0.51	5308376.00	0.51	4703230.50
	0.60	5945233.50	0.60	5242819.00	0.60	4699320.50
	0.68	5850912.00	0.68	7167728.50	0.68	5014424.50
	0.77	5687600.00	0.77	7011890.50	0.77	4882174.00
	0.86	5882010.50	0.86	6251434.50	0.86	4750582.00
	0.94	5972681.50	0.94	5684729.50	0.94	4876558.50
	1.03	7255659.00	1.03	5919862.50	1.03	5002110.50
	1.11	7082748.00	1.11	5986065.00	1.11	4945917.00
	1.20	6978949.50	1.20	5342195.50	1.20	4542485.00
	1.28	6329080.00	1.28	6381928.00	1.28	4551252.00
	1.37	6934855.50	1.37	5305456.00	1.37	5037180.50
	1.45	6384692.50	1.45	6529216.00	1.45	4731332.00
	1.54	6090958.00	1.54	5057578.50	1.54	4867471.00
	1.62	5506279.00	1.62	5554080.50	1.62	4979484.50
	1.71	6496944.50	1.71	5950441.50	1.71	4622257.50
	1.79	6112906.50	1.79	6316059.00	1.79	4022645.75
**Average from 22 scans**		**6467687.27**		**5812949.00**		**4902964.40**

Supplementary Table S2. The intensity of transitions at 22 different run times and the average of the intensity (shown in bold) for *Salmonella* wild-type spiked with 0, 20, 40, 160, 320 nmol of 6-P-F-Asp. S2A: Transition m/z 376→242 of 6-P-F-Asp with collision energy 15 eV. S2B: Transition m/z 376→125 of 6-P-F-Asp with collision energy 15 eV. S2C: Transition m/z 301→216 of spiked internal standard [^{13}C]-F-Asn with collision energy 13 eV.

**Supplementary Table S2A**	**Transition m/z 376→125**									
Spiked 6-P-F-Asp (nmol)	0.00		20.00		40.00		160.00		320.00	
	Run Time (min)	Intensity	Run Time (min)	Intensity	Run Time (min)	Intensity	Run Time (min)	Intensity	Run Time (min)	Intensity
	0.00	0.00	0.00	11192.00	0.00	25376.00	0.00	123008.00	0.00	322560.00
	0.09	0.00	0.09	21988.00	0.09	36128.00	0.09	122575.91	0.09	304922.84
	0.17	0.00	0.17	19032.00	0.17	46880.00	0.17	122168.07	0.17	287305.00
	0.26	0.00	0.26	12254.02	0.26	30448.00	0.26	183805.91	0.26	302627.09
	0.34	0.00	0.34	5484.87	0.34	35191.54	0.34	180206.75	0.34	253275.33
	0.43	0.00	0.43	13808.87	0.43	39935.07	0.43	158146.70	0.43	288241.75
	0.51	0.00	0.51	22123.31	0.51	43280.00	0.51	160397.63	0.51	263957.44
	0.60	0.00	0.60	14118.35	0.60	34904.37	0.60	128195.40	0.60	291384.88
	0.69	0.00	0.69	18136.65	0.69	26538.07	0.69	130131.46	0.69	284086.13
	0.77	0.00	0.77	26074.75	0.77	42084.52	0.77	131985.92	0.77	276862.19
	0.86	0.00	0.86	20694.36	0.86	22509.33	0.86	80984.76	0.86	275865.16
	0.94	0.00	0.94	15878.93	0.94	39552.00	0.94	146022.98	0.94	362865.13
	1.03	0.00	1.03	14783.55	1.03	19094.38	1.03	135327.30	1.03	355287.69
	1.11	0.00	1.11	14326.89	1.11	42003.66	1.11	120180.77	1.11	365177.50
	1.20	0.00	1.20	13378.89	1.20	45120.95	1.20	144740.89	1.20	287131.28
	1.28	0.00	1.28	12464.86	1.28	45692.94	1.28	160453.84	1.28	273737.00
	1.37	0.00	1.37	25889.24	1.37	23269.32	1.37	162622.45	1.37	276840.97
	1.45	0.00	1.45	30609.20	1.45	30125.29	1.45	118524.63	1.45	289878.44
	1.54	0.00	1.54	29273.71	1.54	49431.23	1.54	122331.15	1.54	276631.06
	1.62	0.00	1.62	14662.38	1.62	52248.12	1.62	126078.33	1.62	284511.25
	1.71	0.00	1.71	17610.62	1.71	43485.88	1.71	110225.33	1.71	292463.50
	1.80	0.00	1.80	10192.34	1.80	26444.24	1.80	110794.37	1.80	288238.44
**Average from 22 scans**		**0.00**		**17453.54**		**36351.95**		**135404.93**		**295629.55**

**Supplementary Table S2B**	**Transition m/z 376→242**									
Spiked 6-P-F-Asp (nmol)	0.00		20.00		40.00		160.00		320.00	
	Run Time (min)	Intensity	Run Time (min)	Intensity	Run Time (min)	Intensity	Run Time (min)	Intensity	Run Time (min)	Intensity
	0.00	0.00	0.00	72848.00	0.00	141376.00	0.00	587264.00	0.00	1636352.00
	0.09	0.00	0.09	59200.00	0.09	128200.00	0.09	584831.50	0.09	1625852.88
	0.17	0.00	0.17	82416.00	0.17	115024.00	0.17	582441.88	0.17	1615139.75
	0.26	0.00	0.26	85040.77	0.26	204608.00	0.26	689622.31	0.26	1239408.25
	0.34	0.00	0.34	87669.45	0.34	164315.94	0.34	618482.19	0.34	1431080.13
	0.43	1.19	0.43	96962.67	0.43	124023.88	0.43	600779.81	0.43	1482377.88
	0.51	1525.10	0.51	101496.26	0.51	119136.00	0.51	556385.13	0.51	1280690.38
	0.60	2929.57	0.60	61011.16	0.60	114751.15	0.60	618484.31	0.60	1181678.88
	0.69	1463.28	0.69	120998.69	0.69	110373.60	0.69	611828.75	0.69	1426159.88
	0.77	0.00	0.77	77760.50	0.77	124718.26	0.77	605420.75	0.77	1414211.75
	0.86	0.00	0.86	78286.02	0.86	121741.67	0.86	596523.75	0.86	1294028.25
	0.94	0.00	0.94	90270.34	0.94	118769.88	0.94	615161.44	0.94	1409431.25
	1.03	1.41	1.03	121325.16	1.03	121170.33	1.03	612332.75	1.03	1095200.75
	1.11	656.53	1.11	118351.71	1.11	123588.26	1.11	603494.25	1.11	1544074.38
	1.20	1308.51	1.20	63585.57	1.20	154470.91	1.20	650994.81	1.20	1095740.88
	1.28	1112.59	1.28	119378.38	1.28	138406.86	1.28	645361.69	1.28	1210983.50
	1.37	555.37	1.37	83329.70	1.37	122330.67	1.37	639776.25	1.37	1072302.88
	1.45	3.22	1.45	100493.86	1.45	130143.40	1.45	650522.00	1.45	1210287.63
	1.54	1029.58	1.54	69729.33	1.54	136655.30	1.54	581179.81	1.54	1348132.00
	1.62	0.00	1.62	56662.05	1.62	117541.27	1.62	514583.69	1.62	1360403.00
	1.71	0.00	1.71	59478.60	1.71	131160.92	1.71	583833.50	1.71	1365267.00
	1.80	0.00	1.80	62385.79	1.80	139640.80	1.80	706737.75	1.80	1368735.25
**Average from 22 scans**		**481.20**		**84940.00**		**131915.78**		**611638.29**		**1350342.66**

Supplementary Table S2C		Transition m/z 301→216								
Spiked 6-P-F-Asp (nmol)	0.00		20.00		40.00		160.00		320.00	
	Run Time (min)	Intensity	Run Time (min)	Intensity	Run Time (min)	Intensity	Run Time (min)	Intensity	Run Time (min)	Intensity
	0.00	6759424.00	0.00	7116800.00	0.00	6689792.00	0.00	5249024.00	0.00	4375552.00
	0.09	6878743.50	0.09	6453248.00	0.09	6114872.00	0.09	4684177.50	0.09	4509210.00
	0.17	6997549.00	0.17	6225920.00	0.17	5539952.50	0.17	4119744.25	0.17	4642601.50
	0.26	5800731.00	0.26	6402099.50	0.26	5904384.00	0.26	4612397.00	0.26	4093093.50
	0.34	5214783.00	0.34	6577965.00	0.34	6269012.50	0.34	5125557.00	0.34	4375585.50
	0.43	6196063.50	0.43	6217821.00	0.43	6700971.50	0.43	4525772.00	0.43	4418749.00
	0.51	5990374.50	0.51	6376541.50	0.51	5957632.00	0.51	5136369.50	0.51	4741149.00
	0.60	5958235.00	0.60	6535695.50	0.60	6465635.50	0.60	5126078.00	0.60	4778021.00
	0.68	6575077.50	0.68	5688533.00	0.68	6973179.50	0.68	4506478.00	0.68	4815739.00
	0.77	6554359.00	0.77	5961367.50	0.77	6305606.50	0.77	4424951.00	0.77	4718582.50
	0.86	5731961.00	0.86	6640290.00	0.86	5990072.00	0.86	5015566.50	0.86	4712324.00
	0.94	6674223.50	0.94	6341399.00	0.94	6880552.00	0.94	5021451.50	0.94	4641670.00
	1.03	7052905.00	1.03	6043974.00	1.03	5948986.50	1.03	4916245.50	1.03	4579136.00
	1.11	6861609.50	1.11	6377719.00	1.11	5485464.00	1.11	4451603.50	1.11	4497119.50
	1.20	5900576.50	1.20	6588405.00	1.20	6270440.00	1.20	5055725.50	1.20	4383145.00
	1.28	6817500.50	1.28	6578582.50	1.28	6582760.00	1.28	5155470.50	1.28	4165192.50
	1.37	6336648.50	1.37	6291170.00	1.37	6894370.50	1.37	4608300.00	1.37	4364911.50
	1.45	6035473.50	1.45	6144709.50	1.45	6732421.00	1.45	4711188.00	1.45	4563929.50
	1.54	6368546.00	1.54	5999031.00	1.54	6570569.00	1.54	4947311.50	1.54	4551956.00
	1.62	6490814.50	1.62	6186388.50	1.62	6398089.00	1.62	4723824.00	1.62	4646255.50
	1.71	5918336.50	1.71	6375157.50	1.71	6448500.00	1.71	4759134.00	1.71	4738001.50
	1.79	6152878.50	1.79	6697550.00	1.79	6575024.00	1.79	4634067.50	1.79	4207721.50
**Average from 22 scans**		**6330309.70**		**6355471.23**		**6349922.09**		**4795928.92**		**4523620.25**

Supplementary Table S3. Normalization of transitions from 6-P-F-Asp with respect to the [^{13}C]-F-Asn spiked internal standard. The ratios reported below are the average peak intensity of either transition m/z 376→125 or m/z 376→242 from 6-P-F-Asp relative to transition m/z 301→216 from the [^{13}C]-F-Asn internal standard. Table S3A: Analyses of intensity ratios from data in Table S1 for *Salmonella* wild-type, Δ*fraB*::kan and Δ*fra* island. Table S3B: Ratios determined using different amounts of spiked 6-P-F-Asp (based on primary data in Table S2); these data in turn were used to generate the standard curves in Fig. S2 (6-P-F-Asp spiked *Salmonella* wild-type).

Supplementary Table S3A

	Transition ratios					
	Replicate 1		Replicate 2		Replicate 3	
Transition / Strain used	376→125 / 301→216	376→242 / 301→216	376→125 / 301→216	376→242 / 301→216	376→125 / 301→216	376→242 / 301→216
wild-type	7.96E-06	0.00E+00	2.88E-06	1.37E-06	1.38E-06	4.88E-06
Δ*fraB*::kan	1.41E-02	6.26E-02	1.76E-02	7.04E-02	2.07E-02	7.39E-02
Δ*fra* island	9.28E-07	3.30E-05	5.98E-06	1.20E-05	6.67E-06	5.93E-07

Supplementary Table S3B

	Transition ratios				
Spiked 6-F-Asp (nmol) / Transition	0	20	40	160	320
376→125 / 301→216	0.00E+00	2.75E-03	5.72E-03	2.82E-02	6.54E-02
376→242 / 301→216	7.60E-05	1.34E-02	2.08E-02	1.28E-01	2.99E-01

Supplementary Table S4. The amount of 6-P-F-Asp present in *Salmonella* wild-type, *fraB*::kan and delta *fra* island. While the qualitative trends are similar, there are some differences in the absolute amounts depending on which transition was used for the calculation. Table S4A represents the data computed from transition m/z 376→125 and the standard curve in Fig. S2A, while Table S4B is from m/z 376→242 and the standard curve in Fig. S2B. Two transitions were measured to confirm the overall trends; data from one transition (Table S4B) are shown as Fig. 3, while data from Table S4A are plotted in Fig. S3.

**Supplementary Table S4A**					
	6-P-F-Asp (nmol)				
Strain used	Replicate 1	Replicate 2	Replicate 3	Average	Standard deviation
wild-type	8.54*	8.51*	8.51*	8.52*	0.01
Δ*fraB*::kan	80	110	100	100	10
Δ*fra* island	8.50*	8.53*	8.53*	8.52*	0.01

**Supplementary Table S4B**					
	6-P-F-Asp (nmol)				
Strain used	Replicate 1	Replicate 2	Replicate 3	Average	Standard deviation
wild-type	10.444*	10.446*	10.450*	10.447*	0.002
Δ*fraB*::kan	80	93	89	87	5
Δ*fra* island	10.48*	10.46*	10.45*	10.46*	0.01

*Note: These measurements are below 20 nmol, the lowest concentration used for establishing the standard curve.

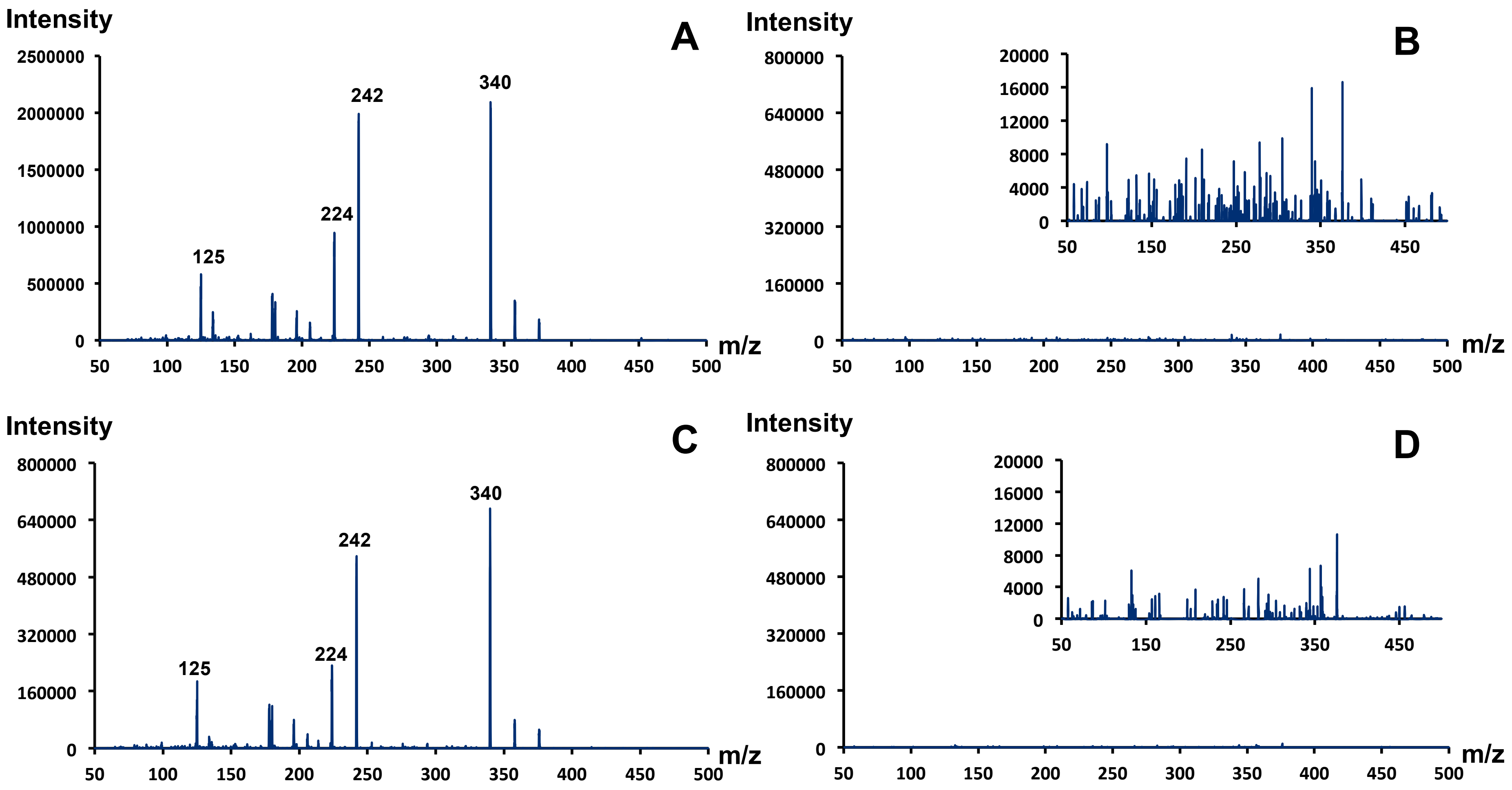

Supplementary Figure S1 Product-ion MS/MS spectra of m/z 376. Collision-induced dissociation (15 eV) of (A) *Salmonella* wild-type (14028) metabolome spiked with 320 nmol of 6-P-F-Asp, (B) *Salmonella* wild-type (14028), (C) Δ*fraB*::kan (HMB206) and (D) Δ*fra* island (HMB215). Inset in panels B and D represents magnification to depict the signal/noise at this level of detection.

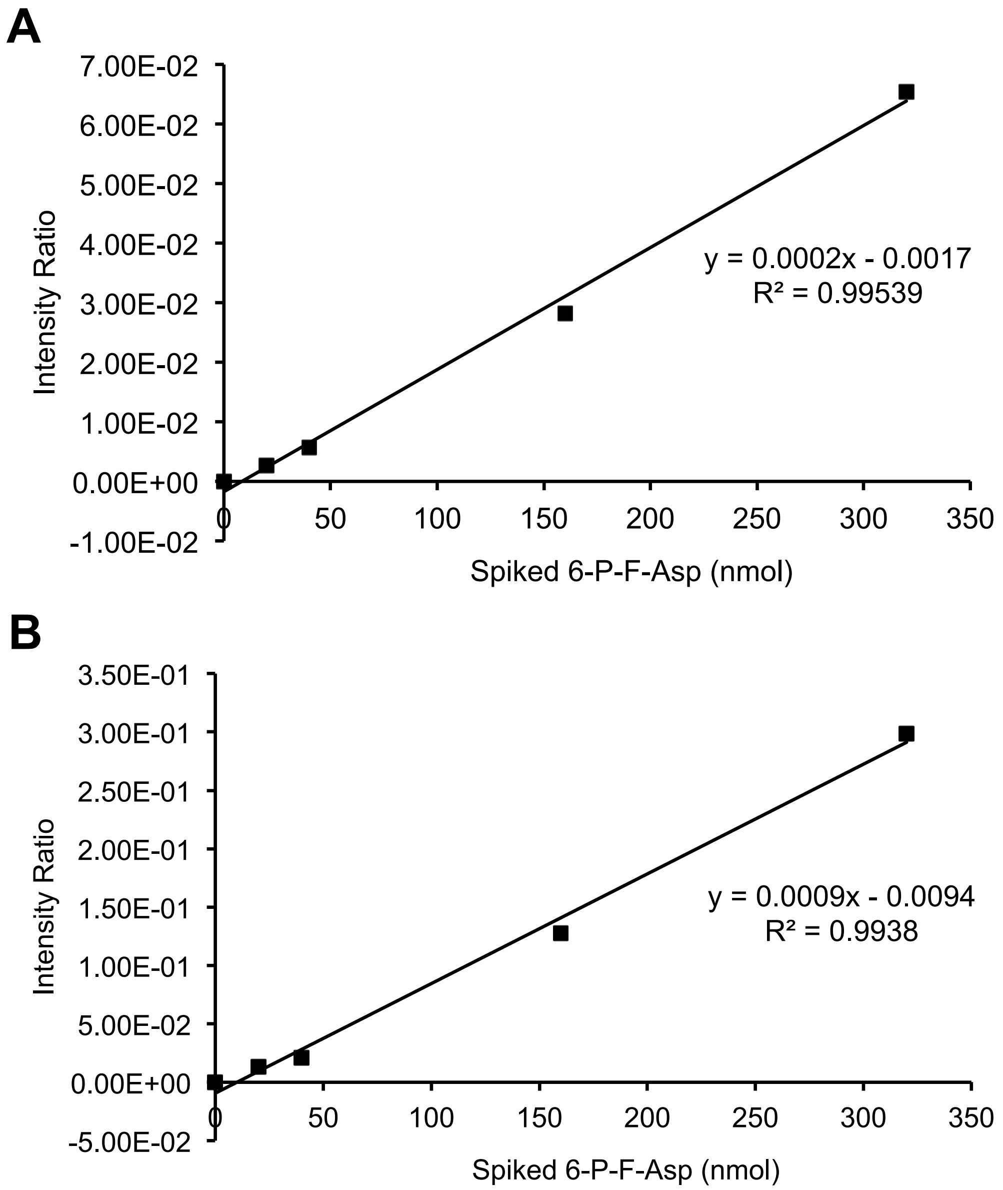

Supplementary Figure S2. Standard curves were constructed by separately plotting the change in peak intensity ratios for two different transitions of 6-P-F-Asp (m/z 376→125, panel A; m/z 376→242, panel B) as a function of the spiked 6-P-F-Asp. These transitions were normalized using the m/z 301→216 from the [^{13}C]-F-Asn internal standard.

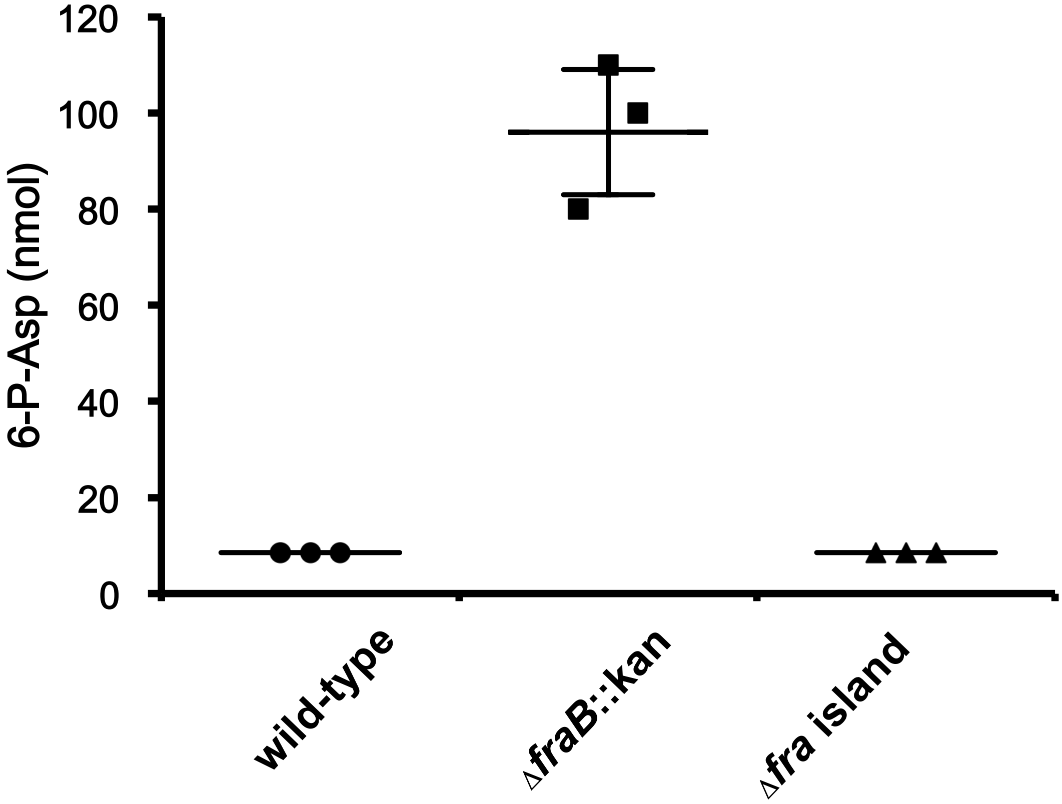

Figure S3. Each point represents mass spectrometry (MS)-based measurement of the levels of 6-P-F-Asp in one biological replicate. Samples were prepared by taking a one-fifteenth aliquot from the cell pellet of a 20-ml culture of *Salmonella* wild-type (14028) or Δ*fraB*::kan (HMB206) or Δ*fra* island deletion (HMB215) mutant. Two transitions were measured to confirm quantitation; data from one transition (Table S4B) are shown as Figure 3, while data from a second transition (Table S4A) are plotted here. The values provided are the mean ± standard deviation from three biological replicates. The lowest concentration on the standard curve was 20 nmol, so the values for wild-type and Δ*fra* island deletion are very low; however, they indicate the reproducible absence of 6-P-F-Asp in these two strains.
